# Biomass Polymer‐Stabilized Hygroscopic Salts in Cellulose Foams for Durable Atmospheric Water Harvesting

**DOI:** 10.1002/smtd.202502418

**Published:** 2026-03-25

**Authors:** Taotao Meng, Bo Chen, Teng Li

**Affiliations:** ^1^ Department of Mechanical Engineering University of Maryland College Park Maryland USA

**Keywords:** atmospheric water harvesting, carboxymethyl cellulose, cellulose, water scarcity

## Abstract

Atmospheric water harvesting (AWH) is a promising strategy to alleviate freshwater scarcity, with hygroscopic salts (e.g., LiCl) widely employed as active sorbents due to their high water affinity. However, practical deployment is hindered by the deliquescence of salts, which can lead to leakage, migration, and potential corrosion. Herein, we report a robust LiCl+CMC@cellulose composite foam fabricated via a scalable room‐temperature impregnation–drying strategy. By introducing carboxymethyl cellulose (CMC) as a polymeric stabilizer, we effectively immobilize LiCl within the cellulose scaffold through ionic coordination, suppressing salt aggregation and leakage. Benefiting from the hierarchical porous architecture of cellulose and this salt immobilization strategy, the optimized composite (treated by 15 wt% LiCl, 0.5 wt% CMC solution) achieves a high water uptake of 3.57 ± 0.15 g at 25°C and 90% relative humidity (RH) within 24 h, corresponding to 16.04± 0.82 g g^−^^1^ when normalized to pristine cellulose foam, representing a 15.4% enhancement over non‐stabilized counterparts. Crucially, the composite exhibits excellent durability, retaining 82.4% of its capacity after ten water uptake–release cycles. Furthermore, a spray‐coated carbon black (CB) surface layer enables rapid photothermal heating (up to 50.6°C under one sun irradiation), facilitating efficient solar‐driven water release. This integrated, environmentally benign design offers a low‐cost, durable platform for scalable, high‐performance AWH.

## Graphical abstract

We fabricate a LiCl+CMC@cellulose composite foam via a scalable impregnation process, enabling excellent AWH water capture‐release cycling stability. The CMC acts as a “molecular glue,” effectively mitigating salt aggregation and leakage. The composite foam delivers a high water uptake of 16.04 ± 0.82 g g^−^^1^ with >80% retention over 10 cycles, while a spray‐coated photothermal layer ensures efficient solar‐driven water release.

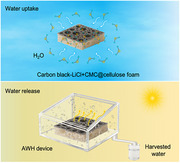

## 1 Introduction

Freshwater scarcity has emerged as one of the most pressing global challenges, necessitating the exploration of water sources beyond conventional groundwater extraction and desalination [1, 2, 3] Atmospheric water harvesting (AWH) offers a decentralized solution by tapping into the approximately 12.9 billion tons of fresh water present in the atmosphere [4, 5, 6]. While condensation‐based systems typically require energy‐intensive active cooling [7, 8, 9], sorbent‐based AWH systems passively capture moisture through specific materials (e.g., hygroscopic salts and hydrogels [10, 11]) and release the absorbed water via solar thermal energy, without the need for sustained external power [12, 13]. Therefore, developing efficient, low‐cost, and environmentally benign sorbents is critical to the practical implementation of sustainable and scalable AWH.

Cellulose‐based foams have gained significant interest as sorbent substrates due to their renewable origin, high porosity, and hydrophilicity [14, 15, 16]. However, while nanocellulose fiber foams offer high specific surface areas, abundant hydroxyl groups, and a highly interconnected porous network, which could facilitate air convection and help gaseous water molecules diffusion in porous structures [17, 18, 19] their fabrication (often involving freeze‐drying or supercritical drying [20]) is costly and challenging to scale [21, 22, 23]. In contrast, microcellulose fiber foams can be fabricated through simple aqueous dispersion followed by ambient drying [24], offering a scalable, mechanically robust, and low‐cost alternative [25, 26]. To enhance water capture, lithium chloride (LiCl) is often impregnated into these foams due to its exceptional hygroscopicity and rapid sorption kinetics [27, 28]. Nevertheless, LiCl suffers from severe deliquescence: upon absorption, it liquefies and leaks from the host substrate, leading to rapid performance decay and potential corrosion [29, 30].

To address these limitations, immobilizing LiCl within polymeric matrices has proven to be an effective strategy for enhancing salt retention and maintaining structural integrity [31, 32]. Existing systems typically employ polysaccharide stabilizers (e.g., alginate, chitosan, and starch) and cellulose‐based carriers such as aerogels, foams, membranes, and wood‐derived scaffolds. Nevertheless, structural degradation and salt loss upon moisture uptake remain persistent challenges. Carboxymethyl cellulose (CMC), a natural anionic polysaccharide derivative, is an ideal candidate. It can form ionic coordination bonds with Li^+^ ions, thereby restricting migration [33]. Moreover, the abundant carboxyl and hydroxyl groups in CMC enable strong interactions with the cellulose network, promoting uniform LiCl dispersion throughout the foam and enhancing the overall mechanical robustness of the composite.

In this work, we fabricate a LiCl+CMC@cellulose composite foam via a scalable impregnation process, enabling uniform LiCl dispersion, robust interfacial bonding, and excellent AWH water capture‐release cycling stability. The CMC acts as a “molecular glue,” effectively mitigating salt aggregation and leakage. The optimized composite foam delivers a high water uptake of 16.04 ± 0.82 g g^−^^1^ with >80% retention over 10 cycles, while a simple spray‐coated photothermal layer ensures efficient solar‐driven water release. By overcoming salt deliquescence and leakage through a dual‐interaction mechanism, this work integrates an unstable liquid sorbent into a durable solid‐state composite, providing a scalable and sustainable pathway for decentralized freshwater generation.

## 2 Results and Discussion

### 2.1 Fabrication and Working Mechanism

**FIGURE 1:**
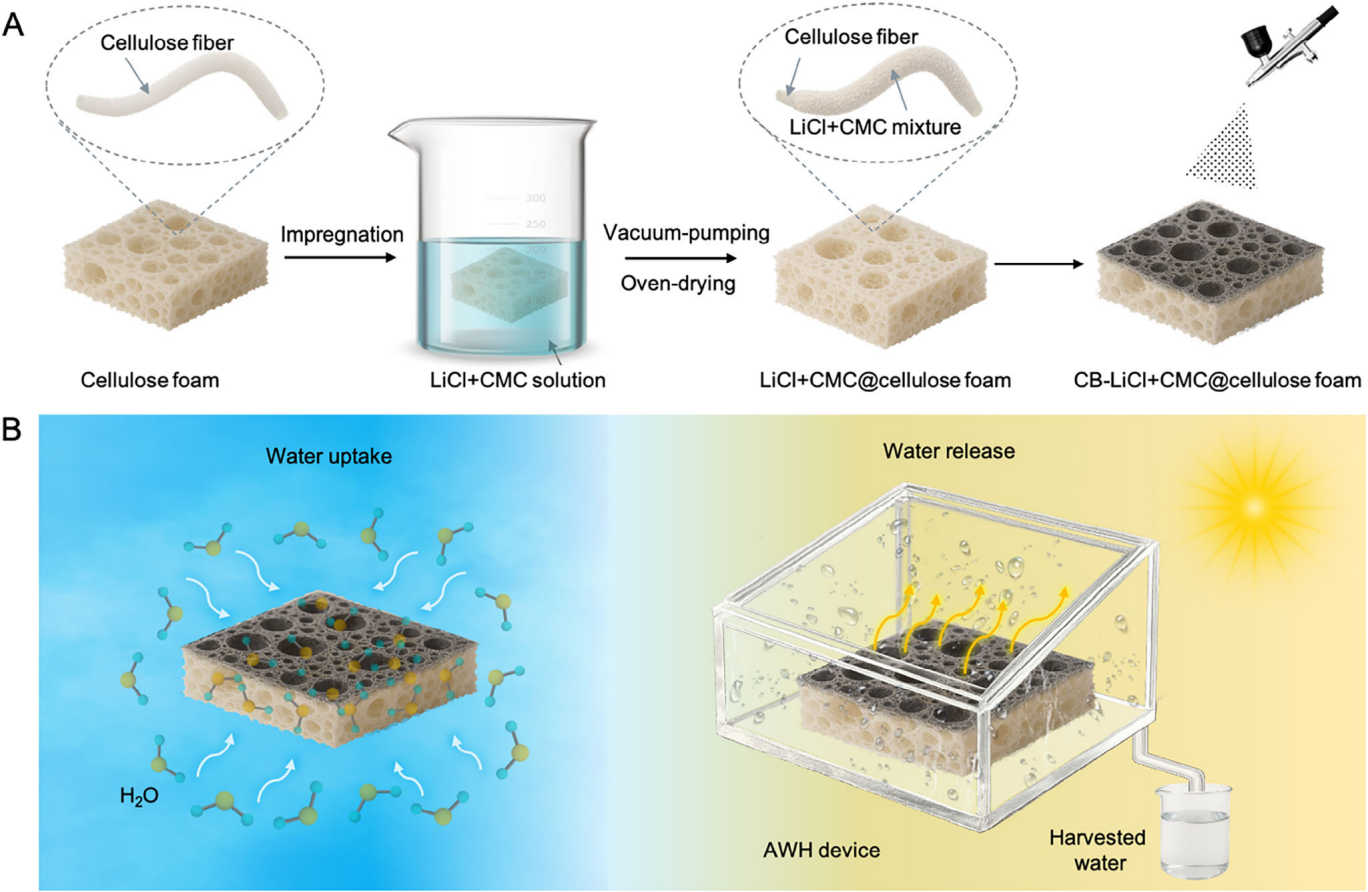
Fabrication and working mechanism of the CB–LiCl+CMC@cellulose foam for atmospheric water harvesting. (A) Schematic illustration of the fabrication process, showing the steps of vacuum impregnation, ambient drying, and CB spray‐coating. (B) Schematic illustration of the AWH mechanism: the hygroscopic foam captures moisture via salt deliquescence, and the CB layer enables solar‐driven water release via photothermal heating.

We design and fabricate a carbon black (CB)–LiCl+CMC@cellulose foam composite that integrates the strong hygroscopicity of LiCl, the stabilizing effect of CMC, and the porous transport architecture of cellulose foam. The composite is produced via a facile, scalable process involving vacuum impregnation, ambient drying, and spray coating (Figure 1A). A commercially available microcellulose fiber foam, derived from low‐cost bleached wood pulp, is selected as the structural scaffold. The foam exhibits a lightweight, highly porous, and hydrophilic 3D fibrous network, providing abundant channels for vapor diffusion while offering sufficient void space for water accommodation.

To evaluate the water uptake performance and structural stability of this microscale foam, we conduct a series of durability and cyclic sorption tests. As shown in Figure S1A, the pristine cellulose foam maintains a stable water uptake capacity over five consecutive water capture–release cycles; the equilibrium water uptake decreases slightly from around 23.71 g g^−^^1^ in the first cycle to approximately 21.45 ± 0.52 g g^−^^1^ (a reduction of ≈9.5%). This excellent recyclability demonstrates that the foam retains its porous architecture and moisture‐handling ability after repeated water capture and release. Furthermore, as illustrated in Figure S1B, the cellulose foam exhibits outstanding structural robustness under various harsh conditions. After soaking (7 d), boiling (6 h), and mechanical blending (6 h at 300 rpm), the mass loss remains extremely low at 1.25%, 1.26%, and 1.07%, respectively. These results confirm that the foam possesses the mechanical integrity and durability required for humid and aqueous environments, making it suitable as a stable, reusable scaffold for the fabrication of hygroscopic composites.

The LiCl+CMC@cellulose composite foam is prepared by dissolving LiCl in an aqueous CMC solution, in which CMC serves as a polymeric matrix to disperse and stabilize the salt via hydrogen bonds and coordinate bonds. The cellulose fiber foam is then immersed in the LiCl+CMC solution, followed by vacuum‐assisted impregnation to promote thorough infiltration of the hygroscopic precursor into the porous matrix. After ambient drying, LiCl and CMC are uniformly distributed and immobilized within the cellulose framework, forming the LiCl+CMC@cellulose composite foam. Finally, a thin CB layer is spray‐coated onto the top surface as a solar‐to‐thermal layer to enable strong solar absorption and efficient photothermal conversion.

Figure 1B illustrates the working mechanism of the CB–LiCl+CMC@cellulose composite foam for AWH. Under humid ambient conditions, the hygroscopic LiCl confined within the cellulose matrix actively captures water vapor from the surrounding air via deliquescence and hydration. In contrast, the porous cellulose network facilitates vapor diffusion and water transport. The incorporated CMC polymer stabilizes the distributed LiCl and maintains the structural integrity during water uptake. Upon solar irradiation, the CB surface layer efficiently converts sunlight into heat, generating a localized temperature rise that accelerates the release of the absorbed water. The released vapor condenses on the inner walls of the transparent chamber and is subsequently collected as liquid water. This water uptake–release cycle enables continuous atmospheric water harvesting driven solely by natural humidity and solar energy.

### 2.2 Morphological Characterization

**FIGURE 2:**
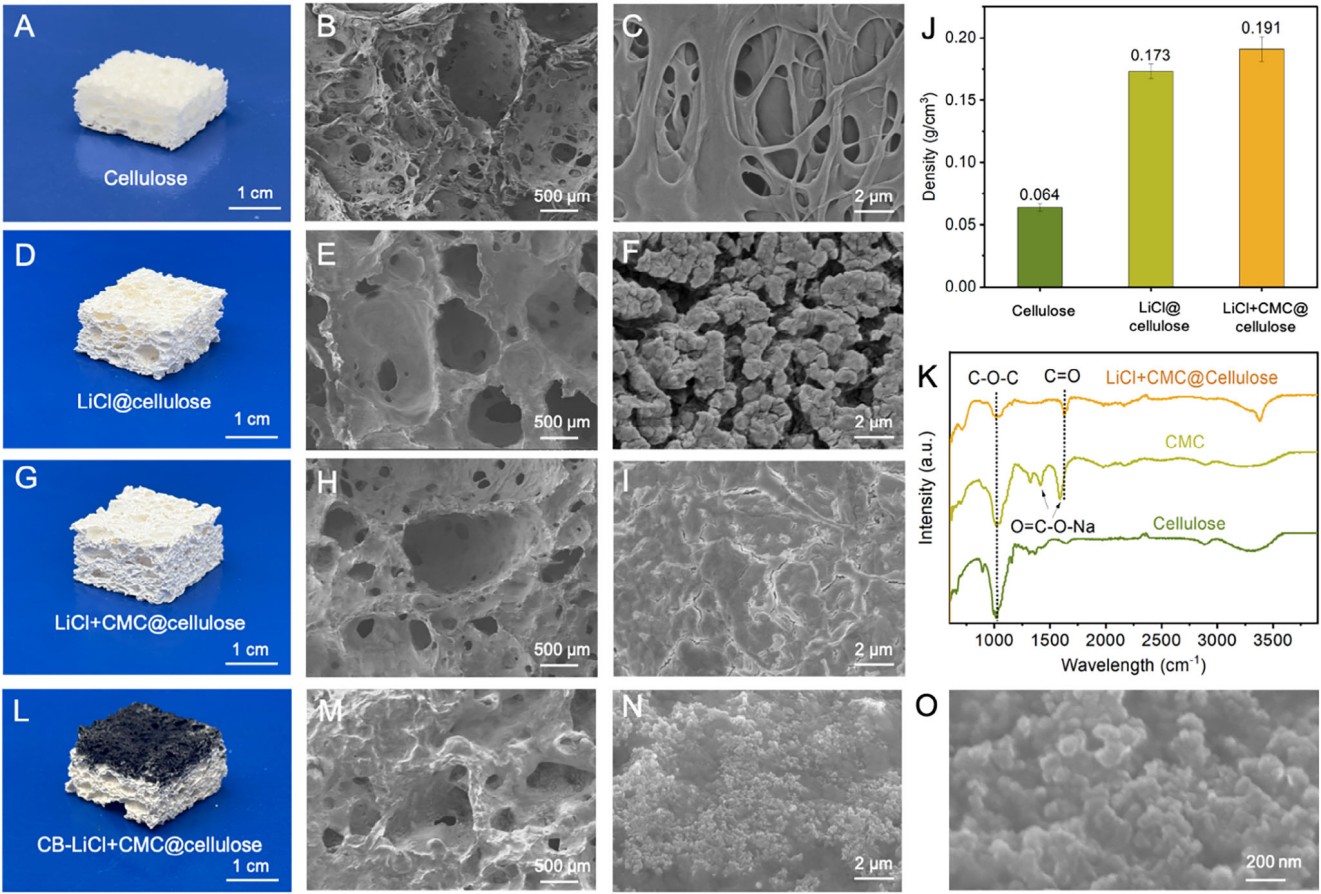
Morphological and structural characterization of LiCl+CMC@cellulose foams. (A–C) Digital photograph (A), low‐magnification SEM (B), and high‐magnification SEM (C) of the pristine cellulose foam. (D–F) Digital photograph (D), low‐magnification SEM (E), and high‐magnification SEM (F) of the LiCl@cellulose foam. (G–I) Digital photograph (G), low‐magnification SEM (H), and high‐magnification SEM (I) of the LiCl+CMC@cellulose foam. (J) Bulk density comparison of pristine cellulose, LiCl@cellulose, and LiCl+CMC@cellulose foams. (K) FTIR spectra of cellulose, CMC, and the LiCl+CMC@cellulose composite, verifying polymer‐salt interactions. (L–O) Characterization of the CB–LiCl+CMC@cellulose foam: digital photograph (L), SEM images (M–O), revealing the uniform distribution of spherical carbon black nanoparticles on the skeleton.

The pristine cellulose foam exhibits a distinctive hierarchical porous architecture, as shown in Figure 2A–C. At the macroscopic scale, the foam displays interconnected open pores ranging from several micrometers to hundreds of micrometers, forming continuous channels that facilitate vapor diffusion and water transport. Scanning electron microscope (SEM) images (Figure 2B,C) reveal that these macropores are enclosed by thin cellulose walls composed of entangled fibrils, creating a 3D fibrous network with abundant micropores. Such a hierarchical porous architecture provides a large specific surface area and excellent hydrophilicity, while also imparting good mechanical flexibility to the foam.

After impregnation with 15 wt% LiCl solution (Figure 2D), the LiCl@cellulose foam, a LiCl‐loaded porous cellulose matrix, appears as a white, porous block with a rougher surface than the pristine sample. The low‐magnification SEM image (Figure 2E) shows that the interconnected porous framework of the cellulose matrix is largely preserved, with macropores of several hundred micrometers still visible, while smaller pores on the order of a few micrometers are partially covered after LiCl loading. At higher magnification (Figure 2F), numerous irregular, island‐like granular deposits are observed on pore walls and fiber surfaces, corresponding to aggregated LiCl crystals formed during drying. The rough and discontinuous surface morphology indicates that LiCl is unevenly deposited on the cellulose surface.

In contrast, the LiCl+CMC@cellulose foam (treated by 15 wt% LiCl, 0.5 wt% CMC solution) exhibits a significantly improved morphology (Figure 2G–I). While the overall pore channels remain well connected, high‐magnification images (Figure 2I) reveal smooth, continuous pore walls with no discernible crystalline particles. Instead, a uniform, dense film‐like coating covers the fiber surfaces. Unlike the island‐like deposition observed in LiCl@cellulose foam, this continuous coating demonstrates that CMC facilitates the homogeneous dispersion and immobilization of LiCl within the cellulose matrix, forming a stable polymer–salt composite layer that enhances interfacial adhesion and structural integrity while preserving the porous framework. Further, XRD analysis was performed to examine the crystalline state of LiCl in the LiCl+CMC@cellulose composite foam. As shown in Figure S2, the XRD patterns of LiCl+CMC@cellulose and pristine LiCl exhibit similar characteristic diffraction peaks, indicating that the crystalline structure of LiCl is preserved after incorporation into the composite foam. Notably, several diffraction peaks in the composite appear sharper, which may be attributed to confined crystallization of LiCl within the polymeric network, leading to more uniform crystallite domains.

Figure 2J compares the bulk densities of the cellulose foam, LiCl@cellulose foam, and LiCl+CMC@cellulose foam. The pure cellulose foam exhibits the lowest density (0.064 g cm^−^^3^), reflecting its highly porous and lightweight structure. After LiCl impregnation, the density of the LiCl@cellulose foam increases to 0.173 g cm^−^^3^, owing to the deposition of LiCl within the pore network. With the addition of CMC, the density of the LiCl+CMC@cellulose foam further increases slightly to 0.191 g·cm^−^^3^, indicating a more compact structure resulting from the formation of a continuous LiCl+CMC coating that bridges cellulose fibers and fills micropores. These results confirm that salt loading and polymer incorporation substantially enhance the structural compactness of the composite foams while maintaining their overall porous architecture.

As shown in Figure 2K, Fourier transform infrared spectroscopy (FTIR) further verifies the formation of polymer–salt interactions within the LiCl+CMC@cellulose composite. The spectrum of pure cellulose exhibits a characteristic C─O─C vibration at 1160 cm^−^^1^, confirming the integrity of the cellulose backbone. For CMC, two signature carboxylate peaks appear at 1590 cm^−^^1^ (asymmetric COO^−^ stretching) and 1430 cm^−^^1^ (symmetric COO^−^ stretching), along with a cellulose‐like C─O─C band near 1100 cm^−^^1^. As shown in Figure K, pristine cellulose exhibits typical C─O─C stretching vibrations around 1030–1100 cm^−^^1^, while CMC shows characteristic carboxylate bands (O═C─O^−^Na^+^) at 1590 cm^−^^1^ (asymmetric stretching) and 1430 cm^−^^1^ (symmetric stretching). After LiCl impregnation, these carboxylate bands slightly shift, accompanied by a pronounced weakening of the C═O stretching peak at 1720 cm^−^^1^. These changes indicate an altered chemical environment of the carboxyl groups, suggesting ionic interaction between Li^+^ and the carboxylate moieties of CMC [34]. Meanwhile, the cellulose C─O─C band at 1160 cm^−^^1^ remains unchanged, confirming the preservation of the cellulose backbone. These spectral changes collectively demonstrate the successful formation of a Li^+^–CMC coordination confined within the cellulose framework, consistent with the morphological observations. To endow the LiCl+CMC@cellulose foam with photothermal properties for solar‐driven water evaporation during the water release process, CB particles are deposited onto the foam surface via a spray‐coating method. Figure 2L–O presents the multiscale morphological characteristics of the CB–LiCl+CMC@cellulose foam. As shown in Figure 2L, the sample exhibits an intact monolithic foam structure, with the upper layer featuring a distinct CB‐modified surface and the lower layer consisting of white cellulose foam. The low‐magnification SEM image (Figure 2M) demonstrates that the surface layer preserves a well‐developed 3D macroporous network. Further observation at higher magnification (Figure 2N) reveals a rough micro‐textured morphology on the pore walls. Notably, the nanoscale SEM image (Figure 2O) further confirms the uniform distribution of spherical CB nanoparticles on the surface of the cellulose skeleton.

**FIGURE 3:**
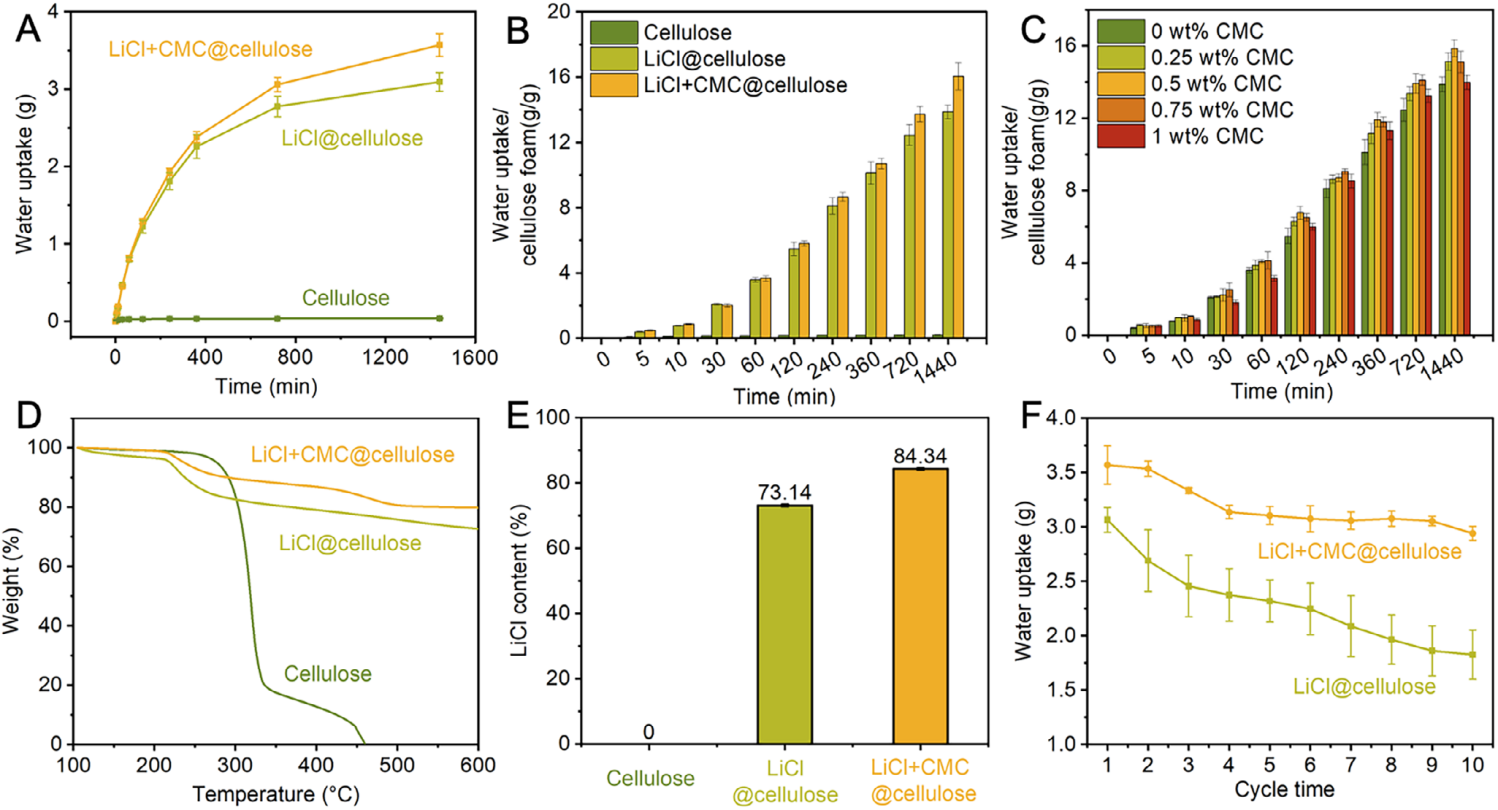
Water uptake performance and stability. (A) Absolute water uptake capacity and (B) normalized water uptake (per gram of cellulose) for pristine cellulose, LiCl@cellulose, and LiCl+CMC@cellulose foams at 25°C and 90% RH. (C) Effect of CMC concentration (0‐1 wt%) on the normalized water uptake capacity of LiCl@CMC@cellulose foams. (D) TGA curves assessing the thermal stability of pristine cellulose, LiCl@cellulose, and LiCl+CMC@cellulose foams. (E) Calculated LiCl content in the different foam composites based on TGA data. (F) Cyclic water‐uptake performance of LiCl@cellulose and LiCl+CMC@cellulose foams over 10 cycles, highlighting the enhanced stability of the CMC‐containing sample.

To further investigate the correlation between structural characteristics and hygroscopic performance, we examine the water sorption behaviors of cellulose, LiCl@cellulose, and LiCl+CMC@cellulose foams. For consistency in comparison, the original cellulose foams used in all tests had similar dimensions of approximately 2 × 2 × 1.2 cm^3^ and a mass of 0.22 ± 0.01 g. As shown in Figure 3A, pristine cellulose exhibited limited moisture uptake capacity at 25°C and 90% relative humidity (RH), reaching only 0.04 ± 0.01 g after 1440 min of water capture. In contrast, LiCl@cellulose captured 3.09 ± 0.12 g of water vapor over 1440 min, indicating that the incorporation of LiCl endowed the composite foam with a high water‐capture capacity. Further addition of CMC resulted in a higher water uptake capacity of 3.57 ± 0.15 g for LiCl+CMC@cellulose, corresponding to an improvement of approximately 15.39% compared with LiCl@cellulose. This enhancement can be attributed to the uniform LiCl dispersion and stronger interfacial bonding facilitated by CMC, which promotes efficient vapor diffusion and water retention within the foam matrix. Figure 3B presents the normalized water uptake per mass of pristine cellulose. The water uptake ratio of LiCl+CMC@cellulose and LiCl@cellulose were 16.04 ± 0.82 g g^−^^1^ and 13.86 ± 0.42 g g^−^^1^, respectively, while pristine cellulose exhibited only 0.22 ± 0.04 g g^−^^1^. The cellulose foam provides a highly porous scaffold that can accommodate large amounts of water. LiCl provides hygroscopic functionality, and CMC further enhances salt dispersion and interfacial compatibility, thereby improving overall sorption performance. To further elucidate the effect of CMC concentration, Figure 3C compares samples with different CMC loadings (treated by 0, 0.25, 0.5, 0.75, and 1 wt% CMC solutions). The 24 h water uptake normalized by cellulose mass first increases and then decreases with increasing CMC content, with the 0.5 wt% CMC sample showing the highest normalized water uptake capacity. The increase could be attributed to the CMC's excellent water retention capacity. When increasing CMC loading in the cellulose foam, excessive CMC may encapsulate LiCl particles, hindering their exposure to humid air and blocking some open pores, thereby reducing effective water uptake.

Thermogravimetric analysis (TGA) confirms the thermal stability and composition of pristine cellulose, LiCl@cellulose, and LiCl+CMC@cellulose under an air atmosphere (Figure 3D). The pristine cellulose exhibits a sharp weight loss in the temperature range of 250–350°C and is almost completely decomposed at around 460°C, corresponding to its typical thermal decomposition behavior [35]. Both LiCl@cellulose and LiCl+CMC@cellulose display significantly higher residual weights at 600°C, indicating the successful incorporation of inorganic LiCl into the cellulose framework. Meanwhile, LiCl@cellulose shows an earlier onset of weight loss at approximately 205°C, which is likely due to the catalytic dehydration effect of LiCl on cellulose [36, 37]. After the introduction of CMC, the weight‐loss behavior of LiCl+CMC@cellulose becomes more gradual, and the residual content is further increased, suggesting enhanced thermal stability [38, 39]. Here, the mass at 205°C is defined as the initial mass of the sample. Since the melting point of LiCl is above 600°C, no decomposition of LiCl occurs below this temperature, and therefore the residual mass at 600°C was regarded as the remaining LiCl content in the composite foam. Based on this definition, the LiCl mass fraction is calculated, and the results are summarized in Figure 3E. The LiCl loading of LiCl@cellulose reaches 73.14±0.31%, while the LiCl content of LiCl+CMC@cellulose is further increased to 84.34±0.25%. To further validate the LiCl loading and retention behavior, inductively coupled plasma–optical emission spectroscopy (ICP‐OES) was employed to directly determine the lithium content in the foam samples before and after cyclic operation. The ICP‐OES‐derived LiCl loading of the as‐prepared LiCl+CMC@cellulose foam reached 82.6 wt%, which is in close agreement with the TGA calculated value of 84.34 wt%, confirming the accuracy of the synthesis strategy and salt incorporation efficiency. In contrast, the LiCl@cellulose sample exhibited a lower initial LiCl content (71.1 wt%), indicating comparatively weaker salt immobilization in the absence of the CMC network. Furthermore, the water‐uptake stability of LiCl@cellulose and LiCl+CMC@cellulose is investigated over 10 water uptake‐release cycles (Figure 3F). In each cycle, water uptake is carried out at 25°C and 90% RH for 24 h, followed by water release via drying in a forced‐air oven at 80°C for 12 h. The water uptake of both materials gradually decreases with increasing cycle number, mainly due to partial LiCl loss during repeated water uptake and release. Nevertheless, recycled‐LiCl+CMC@cellulose (R‐LiCl+CMC@cellulose) consistently maintains a higher water uptake capacity than LiCl@cellulose throughout all cycles. Specifically, the initial water uptake of LiCl+CMC@cellulose is 3.57 ± 0.15 g, and it retains 2.94 ± 0.06 g after 10 cycles, corresponding to 82.4% of the initial capacity. In comparison, the water uptake of recycled‐LiCl@cellulose (R‐LiCl@cellulose) decreases from 3.06 ± 0.11 g to 1.83 ± 0.23 g after 10 cycles, retaining only 59.6% of its initial capacity. After 10 sorption–desorption cycles, the LiCl content in the R‐LiCl+CMC@cellulose foam remained at 70.6 wt%, demonstrating substantial salt retention. However, the R‐LiCl@cellulose showed a more pronounced decrease to 53.9 wt%, suggesting significant LiCl loss during cycling. These results confirm that the CMC matrix effectively immobilizes LiCl within the foam and significantly enhances the cyclic stability of the composite foam.

### 2.3 Optimization of LiCl Loading and Environmental Effects

**FIGURE 4:**
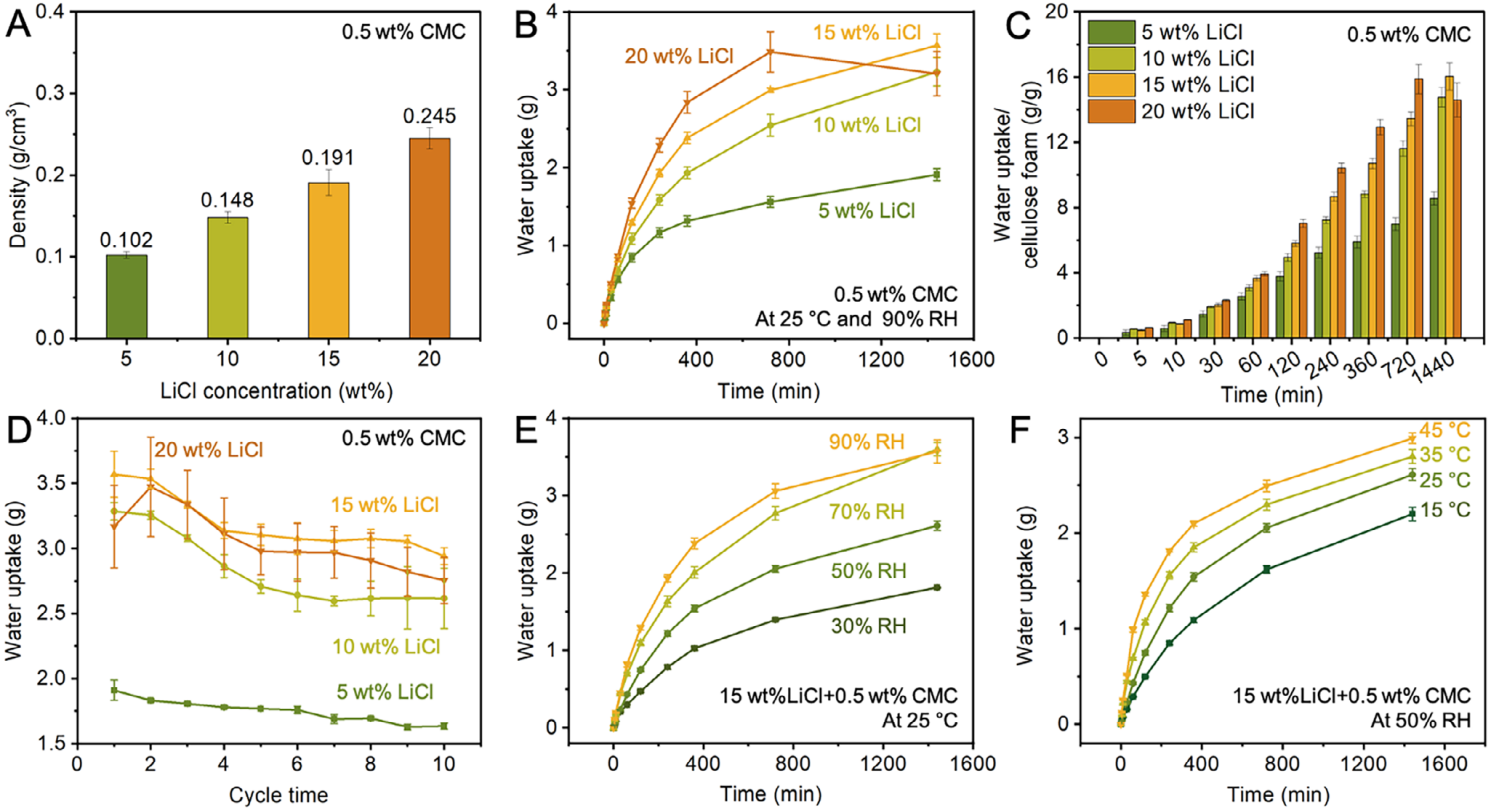
Optimization of salt loading and environmental response. (A) Density of LiCl+CMC@cellulose foams prepared with different LiCl loading (treated by 5–20 wt% LiCl solutions) and a fixed 0.5 wt % CMC solution. (B) Time‐dependent water uptake profiles and (C) normalized equilibrium uptake for LiCl+CMC@cellulose foams with different LiCl loadings at 25°C and 90% RH. (D) Cyclic water uptake stability for different LiCl loadings, showing saturation‐induced decay at 20 wt%. (E) Water uptake of the optimized foam (15 wt% LiCl, 0.5 wt% CMC) at 25°C under varying relative humidity (30–90%). (F) Water uptake of the optimized foam at 50% RH under varying temperatures (15–45°C).

We systematically examine the effect of LiCl loading (treated by 5–20 wt% LiCl with a fixed 0.5 wt% CMC solutions) on the water uptake performance of LiCl+CMC@cellulose foams (Figure 4A–D). The density of the composite foams gradually increases from 0.102 g cm^−^^3^ at 5 wt% LiCl to 0.245 g cm^−^^3^ at 20 wt% LiCl. Figure 4B presents the water‐uptake curves for the various samples at 25°C and 90% RH. To better reflect practical solar‐driven day–night operation, the sorption performance was evaluated within a 12 h (720 min) sorption window corresponding to a typical daytime period. At 25°C and 90% RH, the estimated water uptake at 720 min reaches approximately 1.55 ± 0.07, 2.54 ± 0.13, 2.99 ± 0.02, and 3.48 ± 0.25 g for samples with 5, 10, 15, and 20 wt% LiCl, respectively. Correspondingly, the average water yield rates within 12 h are calculated to be approximately 0.13, 0.21, 0.25, and 0.29 g h^−^^1^, respectively. The 20 wt% sample shows the fastest sorption rate within the first 720 min. After 1440 min, the water uptake reaches 1.91 ± 0.08, 3.23 ± 0.18, 3.57 ± 0.15, and 3.20 ± 0.28 g for 5, 10, 15, and 20 wt% LiCl samples, respectively. The water uptake capacity of the 20 wt% LiCl sample decreases after 720 min, likely due to salt deliquescence exceeding the pore volume, leading to water leakage. Figure 4C compares the equilibrium water uptake capacity normalized by the mass of the cellulose foam at various times. After 1440 min, the normalized uptake increases from 8.56 ± 0.41 g g^−^^1^ for 5 wt% LiCl to 16.04 ± 0.82 g g^−^^1^ for 15 wt% LiCl, while for 20 wt% LiCl the uptake decreases from 15.87 ± 0.91 g g^−^^1^ after 720 min to 14.60 ± 1.04 g g^−^^1^ after 1440 min, owing to the saturation and leakage of absorbed water. The cycling performance shown in Figure 4D indicates that the 15 wt% LiCl sample maintains good sorption stability over ten water uptake‐release cycles, with water uptake capacity decreasing from 3.57 ± 0.15 to 2.94 ± 0.06 g. In contrast, the 5 wt% LiCl sample decreases from 1.91 ± 0.08 to 1.63 ± 0.02 g, and the 10 wt% LiCl sample from 3.28 ± 0.07 to 2.62 ± 0.23 g. For the 20 wt% LiCl sample, the lower initial water uptake (3.16 ± 0.32 g) compared to the second cycle (3.47 ± 0.38 g) is mainly caused by saturation‐induced liquid leakage at high salt loading, followed by a gradual decrease to 2.75 ± 0.18 g after ten cycles. The water‐uptake stability of the high‐LiCl‐content foams can be attributed to the CMC matrix, which forms a robust network that effectively confines LiCl and prevents its migration, thereby reducing LiCl and water leakage and loss. Mechanical stability is critical for maintaining structural integrity and functional performance during water release and regeneration in atmospheric water harvesting applications. All mechanical tests were performed on the LiCl+CMC@cellulose foam (treated by 15 wt% LiCl, 0.5 wt% CMC solution) after equilibration at 25°C and 50% relative humidity for 2 h, ensuring a stable moisture content before testing. The compressive stress–strain behavior of the LiCl+CMC@cellulose foam over a wide strain range (up to 80%) is presented in Figure S3. The foam sustains large compressive deformation with compressive stress reaching the order of 103 kPa at 80% strain. The stress–strain curve exhibits a typical three‐stage response of porous cellulose materials, including an initial elastic regime at low strain, a plateau region associated with progressive pore wall buckling and reversible deformation, and a final densification regime at high strain. This behavior demonstrates the high compressibility and mechanical robustness of the foam under extreme deformation. To evaluate the cyclic compressibility at different deformation levels, cyclic compression–release tests were conducted at maximum strains of 10%, 30%, and 50%, as shown in Figure S4. The foam exhibits stable and well‐defined hysteresis loops during repeated loading–unloading cycles up to 50% strain, indicating effective energy dissipation while maintaining structural integrity. Notably, the loading–unloading curves at each strain level are nearly overlapping, suggesting good mechanical stability without noticeable stress degradation or during cyclic compression–releasing processes. To further examine elastic recovery under moderate deformation, long‐term cyclic compression–release tests were performed at a strain of 30% for 50 cycles (Figure S5). The foam shows near‐linear elastic behavior during loading and minimal residual strain after unloading throughout the cycling process. The stress–strain response remains highly reproducible over repeated cycles, confirming excellent elastic resilience and compressibility under cyclic loading. Overall, the combination of high compressibility at large strain, stable hysteresis behavior under different cyclic strain levels, and excellent elastic recovery over repeated cycles highlights the mechanical robustness of the LiCl+CMC@cellulose foam, which is essential for its durability during repeated compression–release operations in practical applications [40, 41]. We also evaluate the effect of humidity dependence on the water‐uptake performance of LiCl+CMC@cellulose foams (Figure 4E). Water uptake is highly humidity‐dependent. For the LiCl+CMC@cellulose foam (treated by 15 wt% LiCl, 0.5 wt% CMC solution), after the 1440 min water uptake process, the water uptake at 90% RH (3.57 ± 0.15 g) is approximately 2 times that at 30% RH (1.81 ± 0.01 g). As shown in Figure 4F, temperature also affects the water uptake behavior. After exposure to 50% RH air for 1440 min, the water uptake capacity increases from 2.20 ± 0.07 g at 15°C to 2.99 ± 0.06 g at 45°C, which could be ascribed to the higher absolute water content in the air at higher temperature. Overall, a composition of 15 wt% LiCl and 0.5 wt% CMC solution provides the optimal balance, achieving high water uptake capacity and excellent water uptake cyclic stability.

### 2.4 Photothermal Performance and Water Release

**FIGURE 5:**
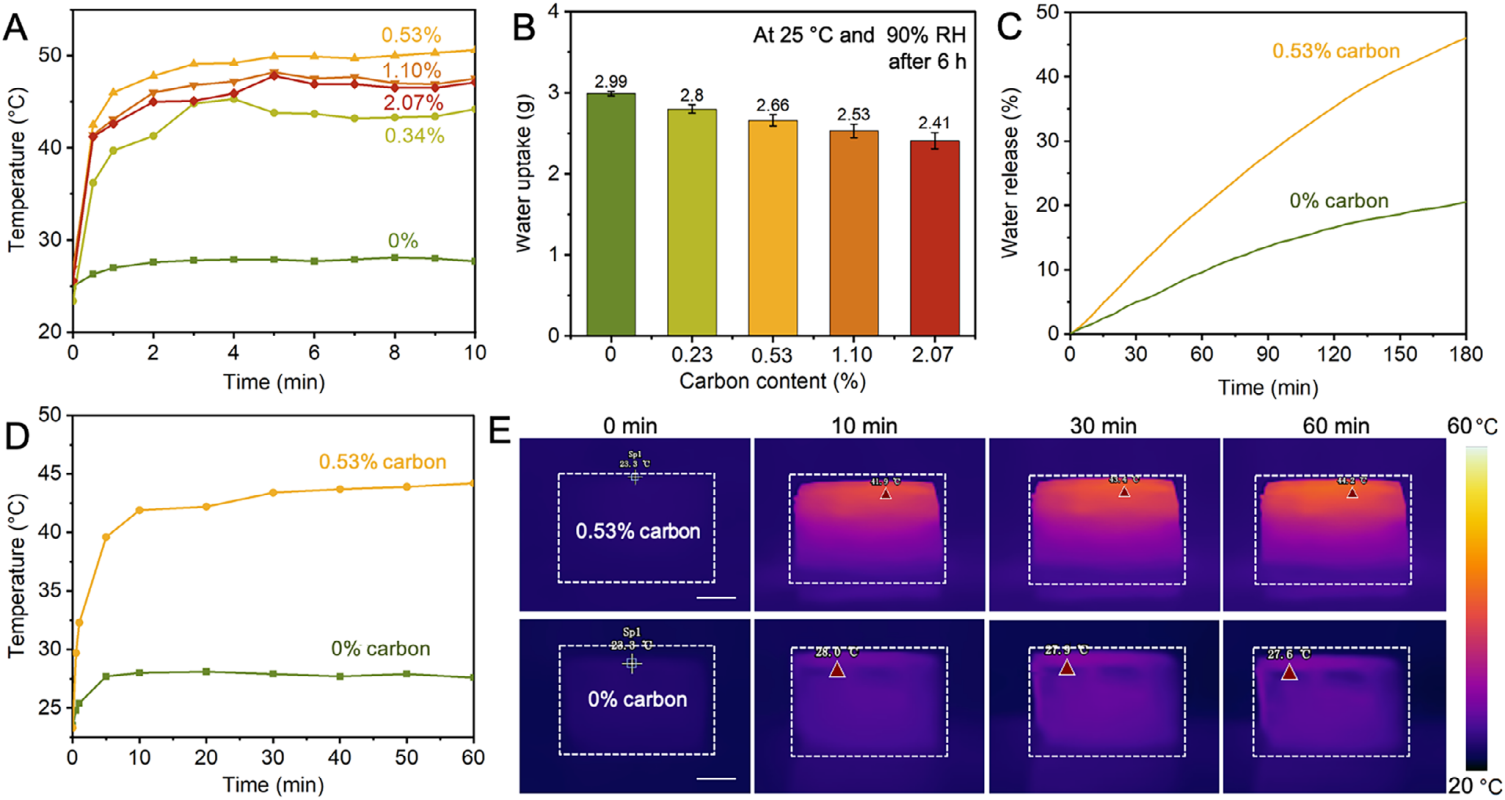
Photothermal performance and solar‐driven water release. (A) Surface temperature evolution of the CB–LiCl+CMC@cellulose foams with different CB contents (0–2.07 wt%) under one sun irradiation. (B) Effect of CB content on water uptake capacity (after six hours at 25°C and 90% RH). (C) Water release kinetics of LiCl+CMC@cellulose foams with and without the optimized 0.53 wt% CB coating under one sun over 180 min. (D) Surface temperature profiles of the coated versus uncoated foams during irradiation. (E) Infrared (IR) thermal images from a side view showing the surface temperature distributions of 0 wt% and 0.53 wt% CB–LiCl+CMC@cellulose foams under one sun irradiation at 0, 10, 30, and 60 mins. Scale bars = 0.5 cm.

To enable solar‐driven water release, we optimize the CB coating on LiCl+CMC@cellulose foams by adjusting the spray time (0, 10, 15, 30, and 60 s) to achieve different carbon contents (0, 0.23, 0.53, 1.10, and 2.07 wt%). The LiCl+CMC@cellulose foam samples (treated by 15 wt% LiCl, 0.5 wt% CMC) are used as the base substrate. Figure 5A shows the evolution of the surface temperature of samples with different carbon contents under one‐sun irradiation (100 000 Lux, i.e., 100 000 Lx). The top surface temperature of the pristine LiCl+CMC@cellulose foam without CB increases from 25°C to 27.7°C within 10 min, indicating that the intrinsic LiCl+CMC@cellulose system exhibits weak photothermal conversion capability. In contrast, the CB‐coated foams show a rapid temperature rise within the first 1 min of irradiation and reach equilibrium after 5 min, demonstrating that the CB coating layer can efficiently convert solar energy into thermal energy to facilitate water evaporation. Notably, the 0.53 wt% CB–LiCl+CMC@cellulose foam exhibits the highest top surface temperature of 50.6°C at 10 min. When the carbon content is further increased to 1.10 and 2.07 wt%, the equilibrium temperatures decrease slightly to 47.5°C and 47.1°C, respectively. This phenomenon may be attributed to the fact that at 0.53 wt%, the CB forms a uniformly dispersed solar‐to‐thermal conversion layer with an appropriate thickness on the foam surface, enabling efficient solar energy conversion and thereby maximizing the local surface temperature. At higher carbon loadings, however, the CB layer becomes thicker and more aggregated, increasing thermal capacity and vertical heat dissipation and slightly reducing the temperature rise at the top surface. As a result, the overall surface temperature becomes marginally lower than that of the 0.53 wt% sample.

However, the CB coating also affects water uptake capacity. Figure 5B shows the mass change of the samples after six hours of water uptake at 25°C and 90% RH. The LiCl+CMC@cellulose foam without a CB layer exhibits a water uptake of 2.99 ± 0.03 g. As the carbon content increases to 0.23, 0.53, 1.10, and 2.07 wt%, the water uptake decreases successively to 2.80 ± 0.05, 2.66 ± 0.07, 2.53 ± 0.08, and 2.41 ± 0.10 g, respectively. These results indicate that the CB coating partially covers and shields the exposed LiCl on the surface, increases the diffusion path length and the resistance to water vapor within the material, thereby reducing the short‐term water uptake capacity. This side effect becomes more pronounced as carbon content increases. Based on these results, the carbon content of 0.53 wt% achieves the optimal balance between photothermal conversion efficiency and water capture performance. It is therefore selected as the optimal choice for the subsequent solar‐driven water release system.

Finally, we test the water‐release performance of the LiCl+CMC@cellulose and 0.53 wt% CB‐LiCl+CMC@cellulose foam under one‐sun irradiation after 6 hours of water uptake, and present the variation in water mass per unit sample mass as a function of irradiation time (Figure 5C). The LiCl+CMC@cellulose foam without CB releases only 20.55% of its absorbed water within 180 min, indicating its limited photothermal conversion capability. In contrast, the 0.53 wt% CB‐LiCl+CMC@cellulose releases 46.04% of the water over the same period, approximately 2.2 times that of the other sample, highlighting that this optimized carbon content enables more effective water evaporation. Figure 5D presents the surface temperature evolution of the LiCl+CMC@cellulose foams without CB (0 wt%) and with a 0.53 wt% CB coating under one sun irradiation for 60 min. The foam without CB shows only a slight temperature increase, rising slowly from an initial 23.5°C to approximately 28°C before reaching a stable temperature plateau, indicating its limited solar energy conversion efficiency. In contrast, the sample coated with 0.53 wt% CB exhibits a markedly enhanced heating rate and a much higher equilibrium temperature. Within the first 1 min of irradiation, its temperature increases rapidly from 23.3°C to 32.3°C, and reaches 39.6°C in 5 min. The temperature continues to rise gradually, reaching 43.4°C at 30 min and finally stabilizing at 44.2°C at 60 min. This pronounced difference demonstrates that an appropriate amount of CB coating effectively improves the material's solar‐to‐thermal energy conversion efficiency, enabling rapid temperature rise and maintaining a high‐temperature steady state, thereby facilitating water evaporation. The IR thermal images (Figure 5E) further provide intuitive visual evidence of the surface temperature evolution during solar irradiation. Consistent with the temperature–time profiles in Figure 5D, the sample containing 0.53 wt% CB rapidly develops a high‐temperature region across the entire surface, indicating effective solar energy conversion and localized heat accumulation. This continuously elevated temperature field contrasts sharply with the 0 wt% CB sample, whose surface remains near ambient temperature throughout the irradiation period, reflecting its limited ability to convert solar energy. The strong correspondence between the IR images and the quantitative temperature measurements confirms that the 0.53 wt% CB coating enables an efficient solar‐to‐thermal layer on the top surface, which is essential for the solar‐driven water release process. To ensure the safety of the harvested water and to verify the absence of salt leakage during operation, inductively coupled plasma–optical emission spectroscopy (ICP‐OES) analysis was performed to quantify the concentrations of Li^+^ and other potentially hazardous metal ions. The calibration curves for all analyzed elements showed excellent linear correlations (*R* ^2^ > 0.99), demonstrating the reliability and accuracy of the ICP‐OES quantification (Figure S6). As shown in Figure S7, the measured Li^+^ concentration in the collected water was 0.023 mg L^−^^1^, which is lower than the U.S. Environmental Protection Agency (EPA) health‐based reference level for Li (0.06 mg L^−^^1^) [42]. Other potentially hazardous metal ions (Pb, Cd, Cr, As, etc.) were either below the detection limit (BDL) or significantly below EPA maximum contaminant levels (MCLs) [43]. These results confirm that the harvested water meets EPA drinking water safety standards, demonstrating effective prevention of salt leakage during cyclic operation.

## 3 Conclusions

In summary, we have successfully developed a robust, all‐biomass atmospheric water harvesting platform by structurally stabilizing hygroscopic salts within cellulose fiber foams. The fundamental innovation lies in the use of CMC as a bifunctional stabilizer: its anionic carboxylate groups for strong ionic coordinate bonds with lithium ions. At the same time, its hydroxyl network adheres firmly to the cellulose scaffold. This dual‐interaction mechanism effectively overcomes the persistent challenge of salt deliquescence and leakage, transforming a typically unstable liquid sorbent into a durable solid‐state composite.

Consequently, the optimized composite (treated by 15 wt% LiCl, 0.5 wt% CMC solution) delivers a superior water uptake of 16.04 g g^−1^ with exceptional cyclic stability, retaining 82.4% of its capacity after ten cycles, significantly outperforming non‐stabilized counterparts. When integrated with a scalable spray‐coated photothermal layer, the system achieves rapid solar‐driven water release with a surface temperature of ≈50.6 °C. By combining low‐cost, sustainable materials with a facile manufacturing process, this work advances a generalizable materials design paradigm of biomass‐based AWH systems by integrating salt stabilization with structural regulation.

## 4 Materials and Methods

Carboxymethyl cellulose sodium (CMC, high viscosity, Sigma‐Aldrich, USA), sodium dodecyl sulfate (SDS, ≥98.5%, Sigma‐Aldrich, USA), plant‐based cellulose foam sheets (Full Circle Plain Jane, USA), acetylene carbon black (99.9%, Alfa Aesar, USA), ethyl alcohol (190 proof, Pharmco by Greenfield Global, USA), and anhydrous lithium chloride (≥98%, Thermo Scientific, UK) were used as received. All chemicals were used without further purification.

### 4.1 Preparation of LiCl+CMC@Cellulose Foams

Commercial cellulose foams were pretreated before use. The foams were washed three times with deionized water to remove surface impurities and residual processing additives. The washed foams were then dried at 80°C for 6 h until their mass stabilized. Afterwards, the foams were cut into blocks measuring 2 × 2 × 0.8 cm^3^ and weighing 0.22 ± 0.01 g, which were used as pristine substrates for subsequent modification. To prepare the 0.5 wt% CMC + 15 wt% LiCl solution, 100 g of deionized water was added to a beaker, followed by the gradual addition of 0.50 g CMC. The mixture was mechanically stirred at 60°C using a mechanical stirrer (IKA RW20 digital mixer, USA) until a clear, homogeneous CMC solution was obtained. Subsequently, a certain amount of LiCl was added, followed by stirring until fully dissolved, yielding a transparent LiCl+CMC mixture. Solutions with different CMC/LiCl concentrations were prepared similarly by adjusting the amounts of CMC and LiCl.

The pretreated cellulose foams were fully immersed in the LiCl+CMC solution and applied in a vacuum chamber (AccuTemp‐19 Vacuum oven 110 V, Across International, USA). A three‐cycle vacuum impregnation was conducted at room temperature, with each cycle lasting 5 min, under a gauge vacuum of approximately 8 kPa, to facilitate deep infiltration of the solution into the foam's hierarchical porous structure. After impregnation, the samples were dried in an 80°C forced‐air oven (KTD‐6000, Zivisamt, USA) for 10 h until their mass reached equilibrium. The obtained samples are referred to as LiCl+CMC@cellulose composite foams.

### 4.2 Preparation of Carbon Black Ink

To prepare the coating ink, a mixed solvent of ethanol and deionized water (mass ratio 1:1) was first prepared by adding 150 g of ethanol and 150 g of water. Next, SDS (0.15 g, ≈0.05 wt%) was added to enhance the wettability and dispersion stability of CB. Then, 3.00 g CB powder was introduced, and the mixture was subjected to ultrasonic treatment using an ultrasonic homogenizer (Model 505, 500 W, 0.5‐inch probe, Fisher Scientific, USA) for 30 min to fully deagglomerate carbon particles and obtain a homogeneous black dispersion. In parallel, 0.30 g CMC was dissolved in 150 g deionized water under ambient‐temperature stirring until a clear, uniform CMC solution was obtained. The CMC solution was then slowly added to the carbon black dispersion under continuous stirring, enabling CMC adsorption onto the carbon particle surfaces. The resulting carbon black ink contained 1 wt% carbon black and 0.1 wt% CMC and exhibited excellent colloidal stability for spray coating.

### 4.3 Preparation of CB–LiCl+CMC@Cellulose Foam

The pretreated LiCl+CMC@cellulose foams were placed on a clean working surface, and the prepared CB ink was spray‐coated evenly onto the foam top surface using a spray gun. During spraying, the distance between the nozzle and the sample surface was maintained at approximately 10 cm, and each spray cycle lasted 10 s. After each cycle, the samples were briefly dried in an oven to promote initial adhesion and coating fixation. Samples with different carbon coating masses were prepared by varying the total spray time to 10 s, 15 s, 30 s, and 60 s, respectively. After spray coating, all samples were dried in a 60°C forced‐air oven for 2 h. The resulting materials are referred to as CB–LiCl+CMC@cellulose foams.

### 4.4 Characterizations

The microscopic morphologies of the foam samples were examined using a Hitachi SU‐70 FEG scanning electron microscope (SEM). Prior to imaging, all samples were coated with a 3 nm gold/Pb layer to improve surface conductivity. Fourier‐transform infrared (FTIR) spectra were recorded at room temperature using a Thermo Nicolet NEXUS 670 spectrometer (USA) with a resolution of 4 cm^−^^1^. A solar simulator (SciSun‐300, Sciencetech Inc., Canada) was used to provide stable and continuous irradiation during the photothermal measurements. The surface temperature evolution of the foams under irradiation was monitored using an infrared thermal camera (225, Fotric Inc., USA). Water uptake experiments were performed under well‐controlled environmental conditions (temperature and relative humidity) using a programmable climate chamber (BINDER MKF056, 240 V, Germany). Thermal stability and decomposition behavior were analyzed using a thermogravimetric analyzer (SDT 650 Simultaneous Thermal Analyzer, TA Instruments, USA). Prior to thermogravimetric analysis (TGA), all samples were vacuum‐dried at 80°C for 2 h to remove residual moisture. TGA measurements were conducted in an oxygen atmosphere with a flow rate of 20.0 mL min^−^^1^, using a heating rate of 10°C min^−^^1^. The samples were first held at 100°C for 1 h, followed by continuous heating up to 600°C to ensure complete combustion of the organic components. The mass recorded at 205°C was defined as the initial mass of the sample (*m*_initial_), thereby excluding the contribution from residual and physically adsorbed moisture. Under these oxidative TGA conditions, the organic cellulose matrix was fully combusted, while LiCl remained as a stable inorganic residue. The LiCl content was calculated according to the following Equation (1):

(1) $$\mathrm{LiCl}\;\mathrm{content}\;\left(\mathrm{wt}\%\right)=\frac{m_{\mathrm{residue}}}{m_{\mathrm{initial}}}\;\times 100\%$$

where *m*_residue_ is the residual mass after TGA under an oxygen atmosphere at 600°C, and *m*_initial_ is the sample mass recorded at 205°C prior to thermal decomposition.

### 4.5 ICP Quantification of LiCl Content

To directly quantify the LiCl loading in the foam samples, inductively coupled plasma (ICP) analysis was performed to determine the lithium (Li^+^) concentration. Dried foam samples were accurately weighed and completely dissolved in deionized water to prepare 1 L of mother solution. Subsequently, 10 mL of the mother solution was diluted to 1 L to obtain the working solution for ICP measurement, resulting in a total dilution factor (DF) of 100.

The Li concentration in the mother solution was calculated as formula (2):

(2) $$C_{\mathrm{mother}}=C_{\mathrm{ICP}}\;\times \mathrm{DF}$$

where *C*_ICP_ (mg L^−^^1^) is the measured Li^+^ concentration in the working solution and DF = 100.

Since the mother solution volume was 1 L, the total lithium mass in the original foam sample was obtained by Formula (3):

(3) $$m_{\mathrm{Li}}=C_{\mathrm{mother}}\;\times V_{\mathrm{mother}}$$

where *V*_mother_ = 1 L. Therefore:

(4) $$m_{\mathrm{Li}}=C_{\mathrm{ICP}}\;\times 100$$

The LiCl mass was calculated based on the stoichiometric relationship between Li and LiCl:

(5) $$m_{\mathrm{LiCl}}=\frac{m_{\mathrm{Li}}}{\left(\frac{M_{\mathrm{Li}}}{M_{\mathrm{LiCl}}}\right)}\;$$

where *M*_Li_ = 6.94 g mol^−^^1^ and *M*_LiCl_ = 42.39 g mol^−^^1^. The mass fraction of Li in LiCl is:

(6) $$\frac{M_{\mathrm{Li}}}{M_{\mathrm{LiCl}}}=0.1637$$

Thus:

(7) $$m_{\mathrm{LiCl}}=\frac{m_{\mathrm{Li}}}{0.1637}\;$$

Finally, the LiCl weight percentage in the foam was calculated as:

(8) $$\mathrm{LiCl}\;\mathrm{wt}\%=\frac{m_{\mathrm{LiCl}}}{m_{\mathrm{foam}}}\;\times 100\%$$

where *m*_foam_ is the initial mass of the dried foam sample.

## Conflicts of Interest

The authors declare no conflicts of interest.

## Supporting information




**Supporting File**: smtd70627‐sup‐0001‐SuppMat.docx.

## Data Availability

The data that support the findings of this study are available in the Supporting Information of this article.
