# Supplementary material for: Biomass Polymer‐Stabilized Hygroscopic Salts in Cellulose Foams for Durable Atmospheric Water Harvesting
Source: Small Methods. 2026 Mar 25;10(9):e02418. doi: 10.1002/smtd.202502418 (PMC13159422; doi:10.1002/smtd.202502418)
Supplement: Supplementary file 1 — Supporting File: smtd70627‐sup‐0001‐SuppMat.docx. [file SMTD-10-e02418-s001.docx]

**Supporting Information**

**Biomass Polymer-Stabilized Hygroscopic Salts in Cellulose Foams for Durable Atmospheric Water Harvesting**

Taotao Meng, Bo Chen^*^, Teng Li^[[1]](#footnote-1)^

*Department of Mechanical Engineering, University of Maryland, College Park, Maryland, USA 20742*


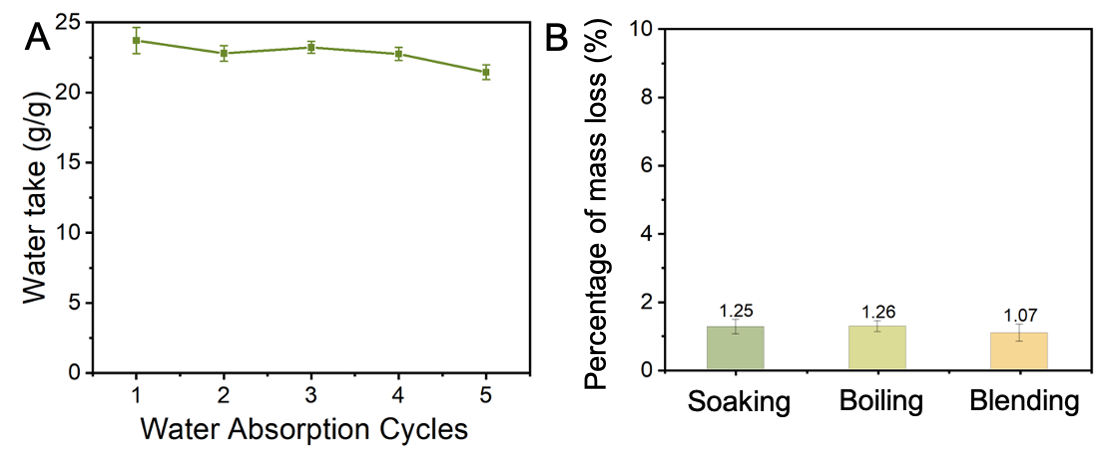


Fig. S1. Water uptake stability and structural robustness of the pristine cellulose foam. (A) Cyclic water uptake performance of the cellulose foam over five consecutive capture–release cycles. (B) Percentage of mass loss of the cellulose foam after soaking for 7 days, boiling for six hours, and mechanical blending for six hours at 300 rpm.

*
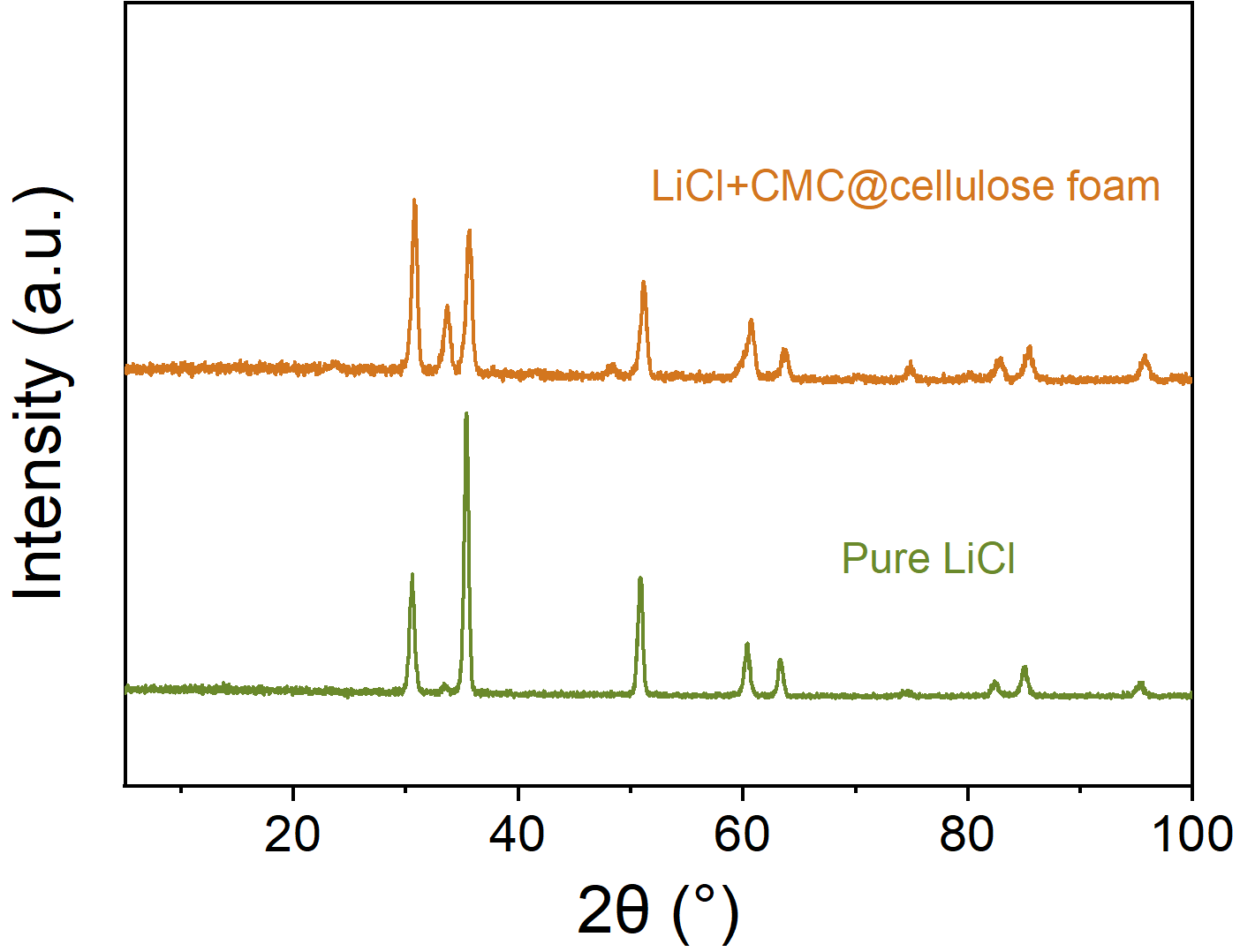
*

Fig. S2. X-ray diffraction (XRD) patterns of pure LiCl, and the LiCl+CMC@cellulose foam composite.


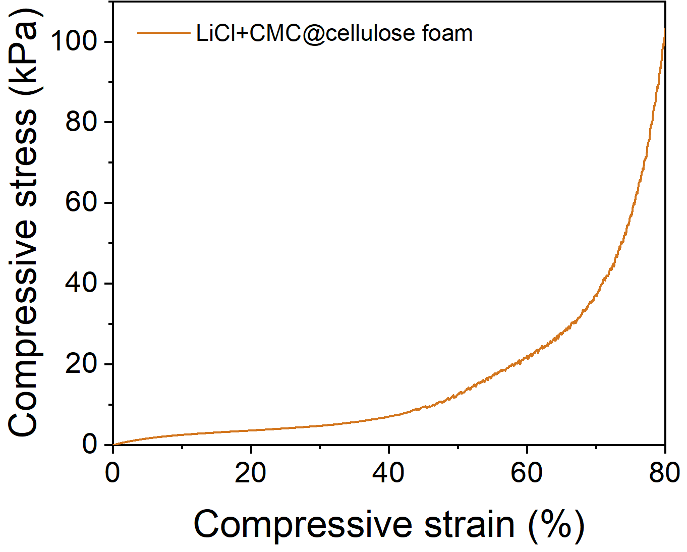


Fig. S3. Compressive stress–strain response of LiCl+CMC@cellulose foam.


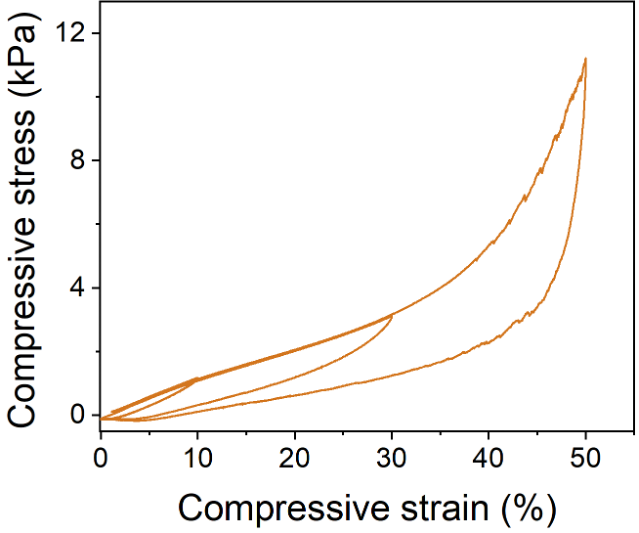


Fig. S4. Cyclic compressive stress–strain behavior of LiCl+CMC@cellulose foam at different strain levels (10%, 30%, and 50%).


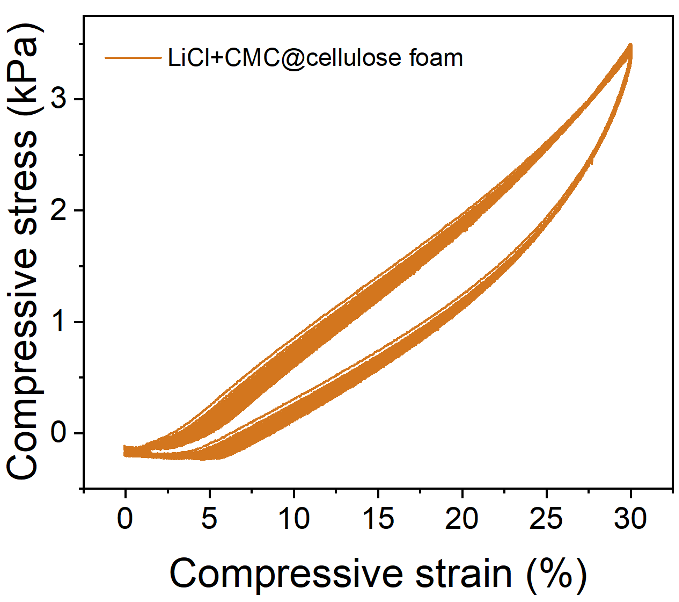


Fig. S5. Cyclic compressive stress–strain behavior of LiCl+CMC@cellulose foam at 30% strain over 50 cycles.


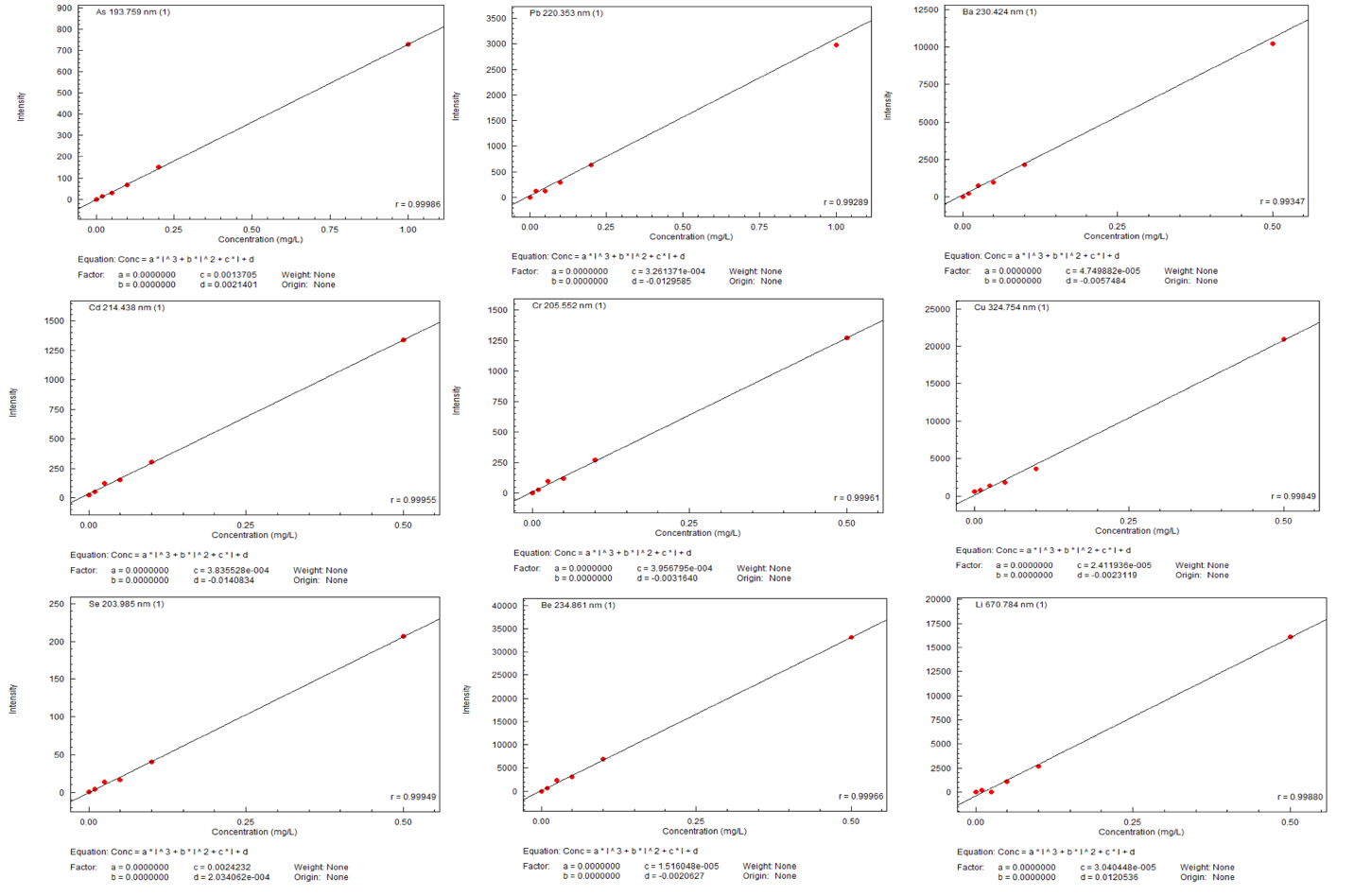


Fig. S6. ICP-OES calibration curves for trace metal ions. Calibration curves of As (193.759 nm), Pb (220.353 nm), Ba (230.424 nm), Cd (214.438 nm), Cr (205.552 nm), Cu (324.754 nm), Se (203.985 nm), Be (234.861 nm), and Li (670.784 nm). All calibration curves exhibit excellent linearity within the investigated concentration range (R² > 0.99).


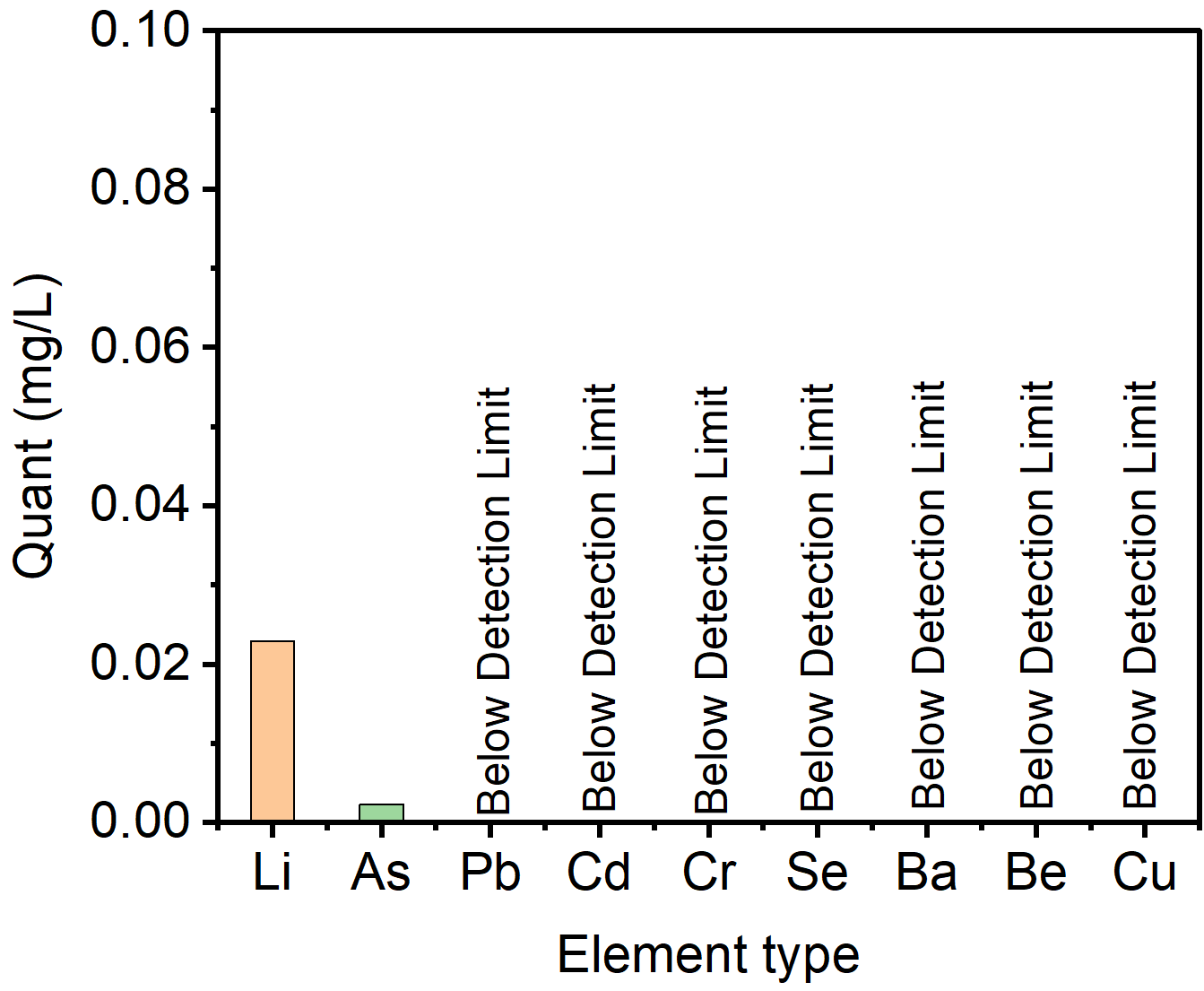


Fig. S7. ICP-OES quantification of ion concentrations in harvested water.

Table 1. ICP quantification of LiCl loading (wt%) in composite foams before and after 10 cycles.

| Sample Name | Original foam (g) | Li+ (mg/L, test solution) | Li in mother solution (mg/L) | Total Li (mg) | LiCl (mg) | LiCl wt% |
| --- | --- | --- | --- | --- | --- | --- |
| LiCl+CMC@cellulose | 0.6072 | 0.821 | 82.1 | 82.1 | 501.5 | 82.6 |
| LiCl+cellulose | 0.335 | 0.39 | 39 | 39 | 238.3 | 71.1 |
| R-LiCl+CMC@cellulose | 0.5835 | 0.674 | 67.4 | 67.4 | 411.7 | 70.6 |
| R-LiCl@cellulose | 0.7596 | 0.67 | 67 | 67 | 409.3 | 53.9 |

1. Corresponding authors. Email: [lit@umd.edu](mailto:lit@umd.edu), [bchen8@umd.edu](mailto:bchen8@umd.edu) [↑](#footnote-ref-1)
